# Managing Chronic Conditions: Lessons Learnt from a Comparative Analysis of Seven Years’ Policies for Chronic Care Patients in Italy

**DOI:** 10.5334/ijic.5686

**Published:** 2022-01-21

**Authors:** Lucia Ferrara, Angelica Zazzera, Valeria D. Tozzi

**Affiliations:** 1 Cergas SDA Bocconi School of Management, Bocconi University, IT

**Keywords:** chronic conditions, policy evaluation, programs, management, multimorbidity, integrated care

## Abstract

This policy paper aims to compare what policies are developed in Italy for the management of chronic patients in order to improve population health, quality of care and patient experience and reduce per-capita cost. The paper also aims to identify the key trends and evolutionary trajectories across the Country.

**Methodology:**

The analysis focuses on 10 Italian Regions and the time span of observation is 7 years (from 2014 to 2020). Data collection and analysis adopts mixed methods in order to have a more in-depth picture of the contextual factors, mechanisms and outcomes. It includes a desk research of the literature and documentary analysis; semi-structured interviews; a theory driven evaluation of 12 programmes identified at the regional level; and a Consensus Conference to discuss and validate the results with an Expert Panel Group.

**Conclusions:**

The paper firstly describes the main policies developed in Italy in the last seven years; secondly, it discusses six main trends and clusters them into three strategies: demand management strategies; strategies to improve the management of comorbid and frail patients; and strategies to improve the coordination between levels of care and the patient journey; thirdly, it discusses eight trends and evolutionary trajectories which are now emerging.

## Introduction

The management of chronic conditions has represented one of the primary targets of the Italian National Health Service (NHS) in the last years, and has it has been one of the main topics on the political agenda. Italy indeed is facing a high burden of chronic conditions mainly related to the aging population (life expectancy at birth in Italy reached 82.7 years in 2015, which is the second highest in Europe after Spain). Data from the Italian Institute of Statistics (ISTAT) reports that around 40% of Italians have at least one chronic disease and 20.9% live with two or more chronic conditions in 2018. Recognising that new types of services are required to meet emerging care needs of chronic patients, a national initiative designed to improve the coordination of chronic care was launched in September 2016. The National Chronicity Plan (NCP - Piano Nazionale della Cronicità; Ministry of Health, 2016) [1] put the spotlight on the topic of chronic disease management and gave an important boost to the implementation of regional policies and programs on chronic diseases. Four years after the publication of the NCP, this policy paper aims to: i) analyse what programs have been developed at the regional level; ii) understand what are the developing trends beyond such programs; iii) share the lessons learned and future directions on chronic disease management.

The Italian experience offers several overarching intuitions that go beyond the current response to the management of chronic conditions and can be useful to inform discussions in other countries and help set future policies.

## Taking charge of chronic patients in Italy – the context

The Italian healthcare system is a highly decentralized, regionally based NHS that provides universal coverage largely free of charge at the point of delivery to all citizens with no restrictions. The NHS is organized into three levels: the State, Regions and local level. While at the national level, the Ministry of Health (supported by several specialized agencies) sets the fundamental principles and the general objectives and goals of the health system, Regional health governments have exclusive authority in execution-level planning and are responsible for organizing and delivering healthcare through a network of population-based ‘local health authorities’ (Aziende Sanitarie Locali, ASLs) and public and private accredited providers.

The Italian healthcare system has undergone great changes since the establishment of the NHS model in 1978. Starting in the ‘90s, the reforms involved a process of decentralization of the NHS, both by delegating considerable managerial autonomy to purchasers and provider organizations and by devolving political and financial authority to the Regions. This high level of autonomy is designed to make the Regions accountable for their performance in achieving general, nationally-set objectives in terms of quantity and quality of services to be guaranteed to all citizens and also to respond effectively to residents’ health needs, given the high heterogeneity of Italian Regions in terms of size, population and levels of economic development.

**Figure 1:**
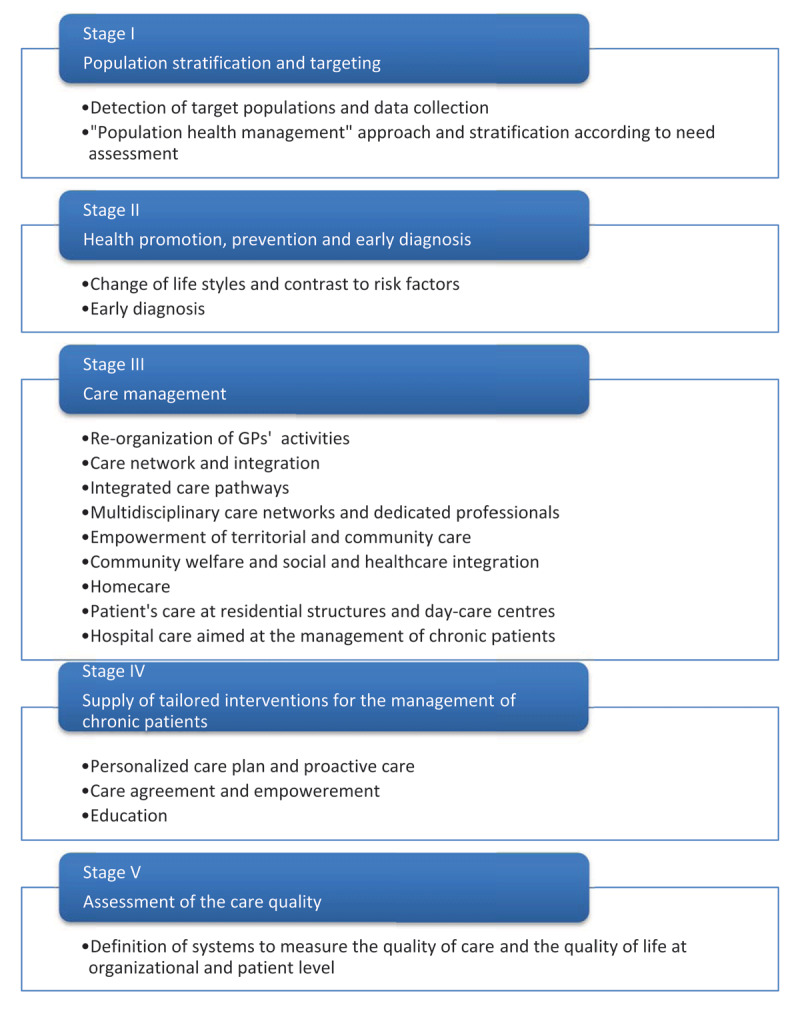
Stages for the management of chronic patients: macro-process and macro-activities [NCP, 2016].

Since 2001, with the reform of Title V of the Constitution Chart, the Ministry of Health remains in charge of setting national standards, goals and strategic directions, while the Regions have further strengthened their positions and started shaping their own systems - in a centrifugal manner. This has led to a high quality, low cost NHS, however with huge differences and gaps in the quality and availability of services among Regions, especially between the northern ones who strengthened their positions and improved overall quality, and the southern ones who began to fall behind, plagued by inefficiencies, a stagnant employment context, and an increasing proportion of the population over 65 years of age, as the exodus of younger residents seeking greater employment opportunities in the North continued [2]. In an effort to improve the management of chronic conditions all over the country, the Italian Ministry of Health defined a National Chronicity Plan (NCP), (2016-18) in 2016 [1]. The Plan stems from the need to harmonize activities in the field at the national level, proposing a document that identifies a common strategic plan and the key elements for the management of chronic conditions (compliance, prevention, homecare, information exchange, education and empowerment, knowledge and competence). The NCP stresses the importance of an integrated, multidimensional, cross-disciplinary, person-centred healthcare system, especially focused on the realization of long-term tailored projects, that aim to improve the quality of life and the experience of patients while containing per-capita costs [3]. The NCP also intends to promote an evolution towards the “Value Based Healthcare” [4]. In this view it describes and analyses in detail the macro process for the management of chronic conditions and it divides the care pathway into different stages. For each chronic condition, the NCP defines general and specific objectives, proposes lines of interventions, expected outcomes and some indicators to monitor, while it leaves the responsibility for the implementation to the regional authorities that are in charge of the execution-level planning and of the organization and delivery of healthcare. The Plan also leaves autonomy to the regional governments to pilot local experiences in order to synergistically maximize and harmonize, from the bottom, successful initiatives and experiments at the national level. ***Figure 1*** gives a summarized description of the macro-activities.

## Methodology

The analysis focuses on 10 Italian Regions (Basilicata, Emilia Romagna, Lazio, Lombardy, Autonomous Province of Trento, Apulia, Sardinia, Sicily, Tuscany and Veneto) selected based on three criteria: firstly, they cover more than 50% of the Italian population; secondly, they grant a wide representative view of the main trends and policies developed throughout the country; and thirdly, each region developed explicit programs for the management of chronic conditions or is implementing piloting projects for some chronic conditions (e.g., Congestive heart failure – CHF). The time span of observation is 7 years: from 2014 to 2020 included therefore, no intervention preceding this time span is reported.

From a methodologic point of view, in order to have a more in-depth picture of the contextual factors, mechanisms and outcomes [5] of the specific programs, we have adopted a multiple case study methodology [6] applying a mixed methods approach [7] by collecting, analysing and mixing qualitative data from different sources. The mixed methods research is particularly suitable for our research due to the complexity in healthcare delivery, as it allows us to explore diverse perspectives and uncover relationships that exist between the intricate layers of our multifaceted research questions. Data collection and analysis have been developed in four steps:

1. First, we ran a desk research of scientific and grey literature, and a documentary analysis of normative sources published during the time of observation. More specifically, the following documents have been analysed: National Chronicity Plan, Regional Chronicity Plans, National Guidelines, Regional Laws, Resolutions, Plans and Programs on Chronic Disease Management, and Regional Strategic Plans.
2. The second step of the analysis consisted of a series of semi-structured interviews to the regional directors responsible for the implementation of primary care and chronic care policies. The objective of such interviews was to validate and integrate the findings of the desk research described in the previews point. Moreover, through the interviews some pilot projects were discussed and some further ongoing policies were explored.
3. The third step of the analysis consisted of the comparative analysis of 12 programs identified at the regional level, by using Chen’s theory-driven evaluation framework [8].
4. Finally, the framework and the outcomes of the comparative analysis were discussed and validated with an Expert Panel Group (EPG) following the Consensus Conference (CC) methodology. The CC is a well-established methodology to define the state of the art of a specific welfare problem or healthcare interventions or policies through an explicit process where information are assessed and discussed by a panel formed by healthcare professionals and other professional, social and institutional experts [9]. The CC aimed at detecting and comparing the choices made at the regional level, and it highlighted the emerging trends and perspectives for the management of chronic conditions. The EPG was made by one representative from each Region, as well as institutional representatives (i.e. the *Italian National Agency for Regional Healthcare Services* (AgeNas), the *National Association of Hospital Cardiologists (*Associazione Nazionale Medici Cardiologi Ospedalieri, ANMCO), the *Italian Federation of Healthcare Organizations and Hospitals* (FIASO, Federazione Italiana Aziende Sanitarie e Ospedaliere), the *National Institute of Health* (ISS, Istituto Superiore di Sanità), and patients’ representatives, i.e. *Italian Association of People with Heart Failure* (AISC, Associazione Italiana Scompensati Cardiaci).

According to the mixed methods’ rational, the following paragraphs discuss both results and conclusions in an aggregate way, without highlighting the distinction among different sources, as the different kinds of collected data provide a better understanding of our research when analysed together, and one single source is not enough to address the aim of our research [7].

## Discussion

### Current trends in the management of chronic conditions

The main interventions in terms of regional plans and programs implemented to improve the management of chronic conditions in the time span taken in consideration (2014 to 2020 included) can be grouped into three macro trends hereinafter described.

**1 Trend: Development of strategies for the identification and management of population health (Demand Management Strategies)**

Assessing the population’s needs in order to plan and provide the supply (possibly in advance or at an early stage) of local health services is a concern that regional and local systems have been facing for many years [10,11,12]. The availability of a huge amount of data have encouraged some Italian Regions to use their own administrative (and sometimes also clinical) databases to develop several Population Health Management (PHM) initiatives that allow them to identify the population, assess their needs and define tailored patient-centred interventions which covers the whole spectrum from prevention to palliative care. This data-driven approach depends on available information, and have been adopted by Emilia Romagna, Lombardy and Veneto Regions [11,12]. Despite the differences, all these PHM initiatives aim to: i) identify the target population based on different characteristics/criteria like geographical location; ii) assess the health of the population using epidemiological data, clinical data, pharmacy and laboratory data, claims data in order to have a “snapshot” of the enrolled population; iii) stratify them into meaningful categories for intervention targeting (i.e. healthy population, population at risk, population with one chronic condition, population with two or more chronic conditions, and frail patients); and iv) define the set of interventions for each risk category from prevention to coordination to palliative care [13]. The approach allows to define in advance the interventions to be carried out by the healthcare organizations based on the needs of the population.

**2 Trend: Strategies to improve the management of comorbid patients**

Effective and efficient long-term management of multiple chronic diseases is one of the greatest health-related challenges facing patients, health professionals, and society. Multimorbidity and comorbidity, indeed, poses substantial difficulties for health care policy and resource allocation decision making [14], as they are often under-evaluated and under-managed, leading to inappropriate drug prescriptions, avoidable hospital admissions, suboptimal care and unnecessary cost overruns [15,16]. Indeed, each multimorbid patient combines different needs and span among different professionals and healthcare services. The effective management of patients with multimorbidities is a key task for the Italian healthcare system, and is forcing it to overcome the single-disease oriented approach and move towards a more comprehensive and harmonized health management system that takes into account patient’s diseases in a global way. In this light, the single integrated care pathways (ICPs) are no longer sufficient; in fact, though they guide the professionals’ behaviours especially from the clinical point of view, they often fail to provide a coordinated and integrated delivery model. The management of a multimorbid patient requires the definition of new organizational models and professional roles that are able to provide an integrated response to the multiple needs of the individual patient and his/her family. This integrator function can be expressed by a variety of models, tools and roles. In each analysed context a central role is played by the General Practitioner (GP), who needs, however, to be organized and integrated into the service chain and, as will be discussed hereinafter, should be supported by automatized (digital?) system of data collection about the patient, a nursing or secretarial support, an automatized system for the access to appliances and aids, etc. Beside the GP, a critical role is played by the healthcare organizations that should support the management of multimorbid patients facilitating their journey within and between services by the development, for instance, of transitional care models (TCM), discharge management models, and nurse transition models.

**3 Trend: Strategies to improve the coordination of the patient journey between levels of care and within services**

The concept of “taking charge of a patient” is often connected to the arrangement of a service supply which is appropriate at a professional level (e.g., standard of evidence-based medicine, EBM), integrated through the use of managerial tools (agreements with GPs, reporting, organizational procedures, etc.) and of integrated care projects that allow the sharing and collaboration among professionals. These highly effective interventions, however, are not sufficient for the management of the most frail and complex population. The management of multimorbid chronic patients requires the definition of new tools or functions that aim to improve the interdependences (e.g. the relations between hospital discharges and the arrangement of nursing homecare services) and the coordination within and between healthcare organizations. The analysis of the regional programs developed within the 10 Italian Regions have revealed a series of initiatives that aim to improve the patient journey across healthcare services, namely, emerging models of patient’s logistics are being processed, not only at an organizational scale but also at an inter-organizational scale. The person who evaluates the case, the one who books the service in advance, the one who verifies the access to services in the appropriate timeframe, the one who transfers the information to the actors’ network can be represented by different subjects, connected by each individual patient’s history. The management of supplying “platforms” appears to be one of the key managerial issues to be faced in the immediate future. This macro trend includes – as a first step - every experience which uses the digital technology, but also those experiences that support the information exchange within the service chain in order to unify and make a synthesis of the care pathways.

### Current regional programs for the management of chronic patients

In order to manage chronic conditions, Italian Regions have used and developed different approaches that can be grouped into 6 macro-categories, namely: PHM, agreements with GPs, Community health centre-based primary care (CHC), intermediate care (IC), transitional care (TC), and eHealth. ***Table 1*** shows the programs implemented in the different regional contexts and their link with the three macro-trends previously described.

**Table 1:** Current regional projects within the 3 macro-trends.


	DEMAND MANAGEMENT		MULTI-MORBIDITY AND FRAILTY		ORGANIZATION OF PATIENT JOURNEY	

REGION	PHM	AGREEMENTS WITH GPS	CHC PRIMARY CARE	INTERMEDIATE CARE	TRANSITIONAL CARE	EHEALTH

Basilicata					X	

Emilia Romagna	X	X	X	X		X

Lazio		X	X			

Lombardy	X	X	X		X	

AP Trento				X		X

Puglia	X	X	X			X

Sardinia			X	X		

Sicily	X		X			X

Tuscany		X	X	X	X	

Veneto	X	X	X	X	X	X

1. PHM approaches are characterized by the identification of the population, the segmentation of the population into subgroups through specific algorithms that allow the grouping of cohorts by health conditions or health needs or risks, the definition of models of care by population target, and the development of monitoring system aimed to evaluate the effects of the intervention. Examples of PHM initiatives at regional level are the Risk-ER Profiles developed in Emilia Romagna since 2017; the Adjusted Clinical Group (ACG) project implemented in Veneto since 2014; the resolution on demand governance with an assessment of the population taken in charge for 64 chronic pathologies in the Lombardy Region. In some cases, the PHM initiatives include predictive models for the use of healthcare resources as it is the case in Emilia Romagna and Veneto. This allows to carry out a case finding of population at risk, for instance, for hospital admission or a clinical complication after a poor compliance with the care pathways. In Lombardy, however, the PHM initiative is only descriptive and allows to identify the population and measure the consumption patterns. Other Regions, like Apulia and Sicily, have recently developed regional databases and repositories and are able to record and link all regional databases at the patient level, which could be considered the pre-requisite for the development of a more structured PHM initiative.
2. Regional and local agreements with the general medicine specifically aimed at taking care of chronic patients are implemented in all Regions, except for Basilicata, the Autonomous Province of Trento, Sicily and Sardinia. The agreements’ focus varies greatly among regions: Emilia Romagna gives a strong orientation towards a GP aggregation in combined forms to improve the quality of physicians’ professional contents; Tuscany enhances the bonds between hospital units and GPs through the development of reference contacts for each territorial functional area (Aree Funzionali Territoriali -AFT); Lombardy regulates the work of GPs through a new chronic care model where they take charge of chronic patients; the Veneto Region develops the Integrated Group Medicine and works to grant an information continuity in the chronicity management.
3. Community health centre-based primary care (CHC) is a public centre where one can find GPs and all patient-centred primary care services. As CHC we refer to all the models introduced in several Regions that go under the definition of Case della Salute, Presidi Territoriali di Assistenza (PTA, *Territorial Healthcare Centres*), Presidi Ospedalieri Territoriali (POT, *Local Hospitals*) and Presidi Socio-Sanitari Territoriali, (PreSST, *Local Healthcare Trusts*). CHC models have been introduced in 8 out of 10 Regions, they concentrate in one single physical space several healthcare services centred on prevention, treatment, rehabilitation, including GP offices, outpatient specialist clinics, social services and some services supplied by the local health authorities - LHAs (e.g., booking service, medical aid management, etc.) and by local authorities (e.g., social worker).
4. Intermediate care (IC) refers to “services or activities concerned with patient’s transitions between hospital and home, and from medical/social dependence to functional independence. It is intended either for post-acute patients requiring recuperative support, or for community dwellers (usually frail or chronically ill) who are at short-term risk of avoidable hospital admission” [17]. IC services have been identified in 50% of the Regions of the sample: Emilia Romagna, Tuscany, Veneto, the Autonomous Province of Trento and Sardinia. The most spread services that has been promoted as type of IC is the Community Hospital, defined by the Ministerial Decree No. 70 of April 2th, 2015 as “a structure with a limited number of beds (15-20) managed by nursing staff, where the medical care is granted by GPs or community paediatricians or other physicians registered with the NHS”. IC services are intended for patient with complex social needs where the objective of care are not primarily medical, and are designed to facilitate the transition from hospital to home, treat chronically or terminally ill people without recourse to hospital care, and prevent long-term institutionalization.
5. Transitional care (TC) refers to “the set of actions designed to ensure coordination and continuity between different care levels and settings within the same structure or between different organizational structures” [18]. TC models are gaining increasing value and widespread, at the international level, to improve the quality and efficiency of health services’ delivery and have been piloted and tested in different Countries as well as within different healthcare service delivery models and organizations. Only Basilicata, Tuscany, Veneto and Lombardy have developed some TC model. Whereas the new models of TC retain the original objectives of discharge management and planning services, their role and organization is changing dramatically within the renewed model of delivery designed by managed care models, and the search for integration across the overall care system from community to acute to end of life care [19]. They are new functions in charge of the healthcare organizations with the aim of facilitating the overall management of patient and of patient’s transition across care settings both ‘step down’ (from hospital into the community) and ‘step up’ (from community care to acute care). TC models often target complex and frail patients with health and social care needs and are especially important for older adults with multiple chronic conditions and complex therapeutic regimens as well as for their family caregiver [20].
6. Several examples of eHealth and telemedicine tool for the management of chronic patients are implemented in Emilia Romagna, Apulia, the Autonomous Province of Trento, Sicily and Veneto. The development of these tools has had a major impulse in 2020 during the Covid-19 pandemic and has represented one of the most innovative modes of taking charge of chronic patients during last year. In 2020 several regions have issued specific resolutions so that public and accredited private providers could remotely provide healthcare services that previously were provided through traditional appointments, and have agreed to allow and recognize televisits at the Regional health system (RHS)’s expenses. eHealth solutions and telemedicine services varies greatly, from disease-specific tools aimed at monitoring some chronic pathologies, such as diabetes, chronic obstructive pulmonary disease (COPD) and heart congestive failure (HCF) like in Emilia Romagna, to the development of apps like the CareWell Project of the Apulia Region or the “@home” project, “Key to Health” or “I prescribe you an app” developed by the Autonomous Province of Trento. In general, many of these initiatives are at pilot stage and are more pathology-specific, since the tools require to be properly tailored on the characteristics of the user (who is often the patient).

All the trends and projects described above are developing in a context of in-depth transformations both in the nature and characteristics of healthcare organizations and the service supply. Italy indeed is witnessing the redesigning of institutional settings in many Regions which often aim at the enlargement of the organization boundaries, the development of CHC primary care models, the development of IC (e.g. community hospitals) [21] and TC models [22].

## Lessons learned from the last 7 years

Based on the analysis of the policies and programs developed in the last seven years in Italy and on the complexity that characterize the subject here treated, the following points should be stressed:

- A great part of the initiatives, programs and models developed recently at the regional level to improve the management of chronic patients target the most complex patients not only from a clinical perspective but also from a social care point of view. It is a need-based target that cuts across all chronic conditions and it is often ascribable to their combination. Examples of programs that focus on this target are the TC model, the CHC based primary care models, and the strategies that go under the umbrella of the PHM.
- A remarkable interest can be observed in the definition of new models of care that make new services available for the whole population and not only for those with specific diseases. There is a tendency towards the definition of a new integrated model of care that is not only disease-specific but go beyond the single disease to take care of the patients as a whole. Examples of this shift can be found either in the development of TC models that support the transition of patients between and within healthcare services, and in the definition of CHC based primary care models that by the concentration and co-location of more professionals and services within the same place try to offer a unified answer to the healthcare needs of the population.
- Where present, disease-specific interventions are aimed at facilitating professional alignment and the adoption of consistent and coherent approaches among different professionals to the same healthcare need. The development of Integrated care pathways (ICPs) or the empowerment of dedicated roles, like the nurse case management, are often the pre-requisite for a correct use of the entire service supply designed for the whole chronic population.
- Finally, an effort is being carried out to create links, bridges and connections between all the healthcare network and to “integrate” all the efforts and the parts of the service supply. TC initiatives follow this direction and specialize a dedicate function which acts as a bridge between different providers, settings and levels of care. Also the CHC based primary care model use the co-location of different professionals and services to integrate all the node of the services within the same location. In this perspective we can also include the PHM initiatives that aim to summarize and link all the information present in the system to provide a unified answer to patients’ needs.

## Conclusion: Evolutionary trajectories

The analysis of the chronic care policies and programs developed in Italy in the last years highlights eight trends and evolutionary trajectories which are now emerging. The following points are not aimed at proposing a reference model, but at representing the most common and cross-cutting choices in the Regions that have committed to implement chronic care policies and models.

**1) The operations management as a future critical issue in the implementation of chronic care policies**

The growing number of chronic comorbid patients forces the system to be aware of the interdependences among different ICPs and to develop tools and resources for analyzing, defining, optimizing and monitoring processes for driving improved performance of interdependent processes. Indeed, the ICPs are structured multidisciplinary care plans which detail essential steps in the care of patients with a specific clinical problem [23], and have been proposed as a way of improving appropriateness, sustainability of patient care and effectiveness. However, the management of multimorbid patients requires to go beyond the single ICP and to align several ICPs, this means to also align “what should be done” with “how it should be done”. How should we avoid to repeat the same laboratory test in a limited period of time for two different pathologies and instead integrate the operational sequences into a unified appointment (one-stop-shop model)? How should we organize the patient’s schedule by booking his follow-up visits on a yearly basis, preventing him from “running after” bookings and accesses? These are just some examples stressing the need to develop the operations management of ICPs and a more comprehensive and harmonized health management system that takes into account patient’s diseases in a global way.

**2) The limits of disease-specific management models and the need of service models focused on chronic conditions**

The analysis has highlighted that the pathologies often subject to regional and local interventions on chronic diseases are traditionally diabetes, COPD and HCF, in order of priority. Diabetes has often been considered the archetype for the development and piloting of new chronic care models. Building on the experiences and lessons learned from diabetic patient’s management, the models developed so far have been gradually extended to other chronic conditions. However, they are facing the problem that the role of the different specialists involved in the care pathway and the management model for other chronic conditions are very different from the diabetic model. Therefore, the supplying models have been continuously adapted according to the distinctive features of the single chronic conditions. It is consequently inferred that we should put aside the model of “one size fits all” chronic conditions. In other words, what works for the management of diabetic patients could not work for other conditions and also the hospital, beside the specialized doctors, could have a role in taking charge of chronic patients, especially those labelled as “highly complex chronic” patients. With highly complex chronic condition we define a complex chronic condition involving multiple morbidities, that requires the attention of multiple healthcare providers or facilities, whose needs are no longer linked to the hospitalization but where specialist competences and expertise still play an essential role.

**3) Unitary organizational responsibility for the fruition of disease-specific services and the spread of clinical responsibilities**

Complex or comorbid patients follow more than one ICP. How is it possible to keep the doctors’ “ownership” in the choice concerning each of the patient’s diseases and to grant the presence of a role or function that can verify if the healthcare pathways at the organizational levels are working, also proposing a support to the patient in the organizational alternatives? This means to find a balance between the competences and expertise that are highly spread according to the healthcare needs of the patient and a unitary responsibility that synthetizes the different perspectives. Though the first issue is largely discussed and organized within cross-disciplinary approaches, for the second one there aren’t many structured examples and organic yet diffused interpretations are unlikely to be seen. The reasons are mainly to be detected in the integration between the operational systems of the different organizations involved in the management of different conditions. The current tools (i.e. information management, inter-organizational agreement, operational procedures for the different services, etc.) are designed according to the service supply and not to the demand made by patients and their families.

**4) Taking charge of the patient means to manage the whole service chain**

Managing chronic patients goes beyond the simple clinical choices. Clinical decisions and commissioning are the elements that qualify the capacity of taking charge of patients in the near future. Commissioning means the ability to guide the patient within the system in order to meet his/her needs in an appropriate, efficient and effective way. Traditionally, this role was in charge of the GP or the local health authority, who were able to guide the patient to navigate the system. However, the recent changes in the configuration of the healthcare organizations, the definition on new model of care and service delivery model (CHC primary care models, intermediate care, community hospital etc.), together with the complexity of managing several chronic conditions at the same time are among the issues that require the definition of new dedicated commissioning functions or roles to support whoever carries out the clinical assessments. This means to go beyond the fence of the single disciplines, to build solid alliances and collaborations among different professionals (horizontal integration), to facilitate the integration between levels of care (vertical integration), and to provide a seamless care pathway.

**5) The responsibility for taking charge of patients (meant as the management of the entire healthcare network) is a process responsibility**

The management of chronic and multimorbid conditions involves multiple professionals, services and levels of care. The complexity of the supplying network, due to the high number of subjects involved, as well as the citizen’s health conditions and his/her expectations, require the definition of new modes of integration between the different parts of the system and the need to guide the patient within the system giving consistent information or, for example, identifying a single and easy point of contact with the patient. This requires the planning of new organizational functions or roles that aim to facilitate the connections between the different providers involved, in order to give the patients more consistent and possibly organized and unified responses. For single chronic conditions this often means to identify the process owner for the different episode of care within the entire care pathway, in other words the care manager, or who is responsible for taking charge of the patient. For multimorbid, complex and frail patients that often present multiple needs (not only clinical but also social needs) and span across different professionals and services, these roles are often not enough to support the transition of patients. Therefore it is necessary to support whoever has the difficult task to “line up” all the interventions for the management of the patient’s condition. Some efforts, in fact, mostly labelled as TC are being made in this direction. At the operational level, this also means to put in place a system that facilitate the information exchange about the patient, the possibility of booking services within the whole service chain, the implementation of forms of combined remote assessment (e.g. telemedicine), or provision of service close to the patient’s home or at the patient’s home.

**6) Growing involvement of patients through specific strategies of patient involvement and co-production forms**

The high number of chronic patients and the complexity of their needs require a greater involvement of patients and patient representatives. Indeed, the benefit of patient participation have been investigated in many studies and include, among others, improved outcomes increased patient satisfaction, increased trust in services due to increased freedom, knowledge and transparency [24]. Based on this, specific strategies of patient involvement in decision-making process should be developed by healthcare organizations. This means to identify and develop specialized competences and tools able to support a structured dialogue with patient representatives within the healthcare organizations, to make evident what the single organization is able to put in place for the different populations but also to collect the users’ feedback on a structured and cyclical basis, in order to consistently improve the care process. The capacity of the healthcare organizations to dialogue and interact with the stakeholders (the most relevant of whom are citizens and patients) can also involve other forms of co-production (e.g. information about the service network and access conditions, or even production of additional services). This process requires active collaboration by the consumer (patient) and the producer to ensure quality and enhance value.

**7) The service formulae ascribable to the Community health centre-based primary care (CHC) represent the central gateway for: a) guiding the patient; b) supplying integrated services; c) granting the access to local service network**

Forms of physical concentration of services and co-location ascribable to the CHC model already exist or are at the design stage in many contexts. These models support the community building processes by informing the community about the existing service available and moving the supplying offer close to the place where the patient lives. It is a substantial evolution of local service supply where multiple professional skills and the patient’s logistics result into some distinctive features: 1) the possibility to have GPs operating in the same physical place of specialized doctors and administrative staff allows the access to specialist competences largely diffused in a physical place where professional relationships are easy and helps the exchange among professionals with different roles intervening in the response to the same patient; 2) also administrative issues can found a response (e.g. possibility to book a service, request of aids and appliances).

**8) Increase the scale and adoption of Population Health Management approaches**

We stress the need to read and interpret on a periodical basis not only the evolution of disease prevalence and incidence, but especially the consumption patterns of the different sub populations. This information is essential not only to guide the service planning (after checking the conditions of its appropriateness) but also to support the commission role by the public subject to the different healthcare suppliers (e.g. assessment about quality, consumption patterns, appropriate procedures and performances that qualify diagnosis, treatment and follow-up according to evidence). Other issues might improve the capacity to know what citizens consume when they experience some disease, i.e. the possibility to link social information about the patient and his family, his housing conditions and his socio-demographic features (from education to age). Another issue is the possibility to know the mix between public and private consumptions (either intermediated or not) supported by the patients and their families. Even if these reflexions find large consensus forms, they are in contrast with some remarkable limitations that – though legitimate – restrict their developmental spaces (one of the strictest ones concerns the privacy management).
